# Retinoid X Receptor Agonists Upregulate Genes Responsible for the Biosynthesis of All-*Trans*-Retinoic Acid in Human Epidermis

**DOI:** 10.1371/journal.pone.0153556

**Published:** 2016-04-14

**Authors:** Lizhi Wu, Sandeep C. Chaudhary, Venkatram R. Atigadda, Olga V. Belyaeva, Steven R. Harville, Craig A. Elmets, Donald D. Muccio, Mohammad Athar, Natalia Y. Kedishvili

**Affiliations:** 1 Department of Biochemistry and Molecular Genetics, University of Alabama at Birmingham School of Medicine, Birmingham, AL, 35294, United States of America; 2 Department of Dermatology, University of Alabama at Birmingham School of Medicine, Birmingham, AL, 35294, United States of America; 3 Department of Chemistry, University of Alabama at Birmingham, Birmingham, AL, 35294, United States of America; Laboratoire de Biologie du Développement de Villefranche-sur-Mer, FRANCE

## Abstract

UAB30 is an RXR selective agonist that has been shown to have potential cancer chemopreventive properties. Due to high efficacy and low toxicity, it is currently being evaluated in human Phase I clinical trials by the National Cancer Institute. While UAB30 shows promise as a low toxicity chemopreventive drug, the mechanism of its action is not well understood. In this study, we investigated the effects of UAB30 on gene expression in human organotypic skin raft cultures and mouse epidermis. The results of this study indicate that treatment with UAB30 results in upregulation of genes responsible for the uptake and metabolism of all-*trans*-retinol to all-*trans*-retinoic acid (ATRA), the natural agonist of RAR nuclear receptors. Consistent with the increased expression of these genes, the steady-state levels of ATRA are elevated in human skin rafts. In ultraviolet B (UVB) irradiated mouse skin, the expression of ATRA target genes is found to be reduced. A reduced expression of ATRA sensitive genes is also observed in epidermis of mouse models of UVB-induced squamous cell carcinoma and basal cell carcinomas. However, treatment of mouse skin with UAB30 prior to UVB irradiation prevents the UVB-induced decrease in expression of some of the ATRA-responsive genes. Considering its positive effects on ATRA signaling in the epidermis and its low toxicity, UAB30 could be used as a chemoprophylactic agent in the treatment of non-melanoma skin cancer, particularly in organ transplant recipients and other high risk populations.

## Introduction

All-*trans*-retinoic acid (ATRA) is a highly potent derivative of vitamin A that is required for virtually all essential physiological processes and functions because of its involvement in transcriptional regulation of over 530 different genes [1–3]. ATRA exerts its actions by serving as an activating ligand of nuclear retinoic acid receptors (RARα-γ (NR1B1-3)), which form heterodimers with retinoid X receptors (RXRα-γ (NR2B1-3)) [4]. In addition to RARs, RXRs can heterodimerize with numerous other nuclear receptors, including vitamin D receptor (VDR (NR1I1)), thyroid hormone receptor (TR (NR1A1-2)), liver X receptor (LXR (NR1H1-2)), and peroxisome proliferator-activated receptor (PPAR (NR1C1-3)). In heterodimers with permissive partners (e.g., PPAR and LXR), RXR can be activated by RXR agonists in the absence of the ligand of the partner, and when both partners are activated, they act in an additive or synergistic manner. In contrast, in conditionally permissive RXR/RAR heterodimers, RAR and RXR agonists together can have a greater effect on gene transcription than the RAR agonist alone, but the ligand-dependent transcriptional activity of RXR appears “subordinated” to the binding of ATRA to its RAR partner [5]; hence, the cellular levels of ATRA are critically important for the activation of RXR/RAR signaling.

As reviewed in detail previously [6], the concentration of ATRA in the cells is tightly controlled by the enzymes responsible for its biosynthesis and degradation. ATRA is produced from all-*trans*-retinol in two oxidative steps: first, retinol is oxidized to retinaldehyde by retinol dehydrogenases, and then retinaldehyde is oxidized to ATRA by retinaldehyde dehydrogenases [6]. The first step is reversible and is controlled by the opposing activities of retinol dehydrogenase(s) and retinaldehyde reductase(s) [6]. A recent study demonstrated that the retinol dehydrogenase 10 (RDH10, SDR16C4) and the ATRA-inducible retinaldehyde reductase DHRS3 (dehydrogenase/reductase 3, SDR16C1) mutually activate each other [7], indicating that the rate of ATRA biosynthesis is regulated *via* a sophisticated mechanism, possibly involving RDH10/DHRS3 protein-protein interactions. The levels of ATRA are also controlled *via* ATRA-inducible cytochrome P450 enzymes (e.g., CYP26A1 and CYP26B1), which inactivate ATRA by hydroxylation [6].

The availability of retinol for ATRA biosynthesis is determined by the uptake and retention of retinol in the cells. The uptake of retinol is believed to be mediated by “Stimulated by Retinoic Acid gene 6” (STRA6), a membrane receptor for plasma retinol binding protein 4 (RBP4) (reviewed in [8]), which delivers retinol to peripheral tissues from liver. Lecithin:retinol acyltransferase (LRAT) converts retinol to its storage form, retinyl esters (reviewed in [9]), which can be hydrolyzed back to retinol by retinyl ester hydrolases when needed. Thus far there is no evidence that retinyl ester hydrolases are regulated by vitamin A status, even though ATRA induces expression of both *LRAT* and *STRA6* genes [10, 11].

It is well established that disruption of normal ATRA signaling is associated with numerous pathophysiological changes leading to carcinogenesis, impaired immune function, and metabolic dysregulation [12–16]. Skin is one of the major targets of ATRA signaling [17], where it is essential in the regulation of several aspects of skin cell proliferation, differentiation, apoptosis, and epidermal barrier function. Alterations in retinoid metabolism, signaling and concentrations have been observed in various dermatoses, such as psoriasis [18], ichthyosis [19], and in atopic dermatitis [20]. Treatments with ATRA or synthetic RAR agonists appear to ameliorate some of these conditions. For example, acitretin and tretinoin were shown to be effective for the treatment of actinic keratoses and to delay the development of SCCs in patients with xeroderma pigmentosum, a disease in which there is an inherited predisposition to ultraviolet-induced cancer [21, 22]. Systemic retinoids have also exhibited a chemoprophylactic effect in the treatment of non-melanoma skin cancer, particularly in organ transplant recipients and other high risk populations [23–25]. Unfortunately, because these agents must be continued indefinitely to maintain their protective benefits, the use of retinoids is limited due to their teratogenic potential and other intolerable adverse effects, including hypertriglyceridemia, mucocutaneous inflammation and hepatotoxicity.

As an alternative approach, several laboratories developed retinoids that bind selectively to the RXR component of RXR/RAR heterodimers (known as rexinoids). As summarized in several review articles, the identity of endogenous RXR agonists remains controversial [26–28]. The first candidate for this role, a pan-agonist 9-*cis*-RA, was originally identified as endogenous RXR ligand through the reverse endocrinology approach (reviewed in [29]). However; subsequent analysis showed that 9-*cis*-RA is detectable at physiologically relevant levels only in pancreas [30]. In brain, docosahexanoic acid was proposed to serve as the endogenous ligand for RXR [31]. However, the physiological relevance of docosahexanoic acid has been questioned considering its limited availability in unesterified form [32, 33]. In addition, phytanic acid has been proposed but not yet proven to serve as endogenous RXR ligand *in vivo* [34, 35]. A recent study identified 9-*cis*-13,14-dihydroretinoic acid as the endogenous agonist of RXRs based on reduced RXR signaling in RBP1-null mice, which had decreased levels of 9-*cis*-13,14-dihydroretinoic acid in serum, liver, and brain [36]. In support of physiological relevance of 9-*cis*-13,14-dihydroretinoic acid as the RXR ligand, supplementation with this compound improved memory performance of *Rbp1*^-/-^ mice. Another recent study showed that β-apo-13-carotenone, an eccentric cleavage product of β-carotene, antagonized the activation of RXRα by 9-*cis*-RA by inducing the formation of the "transcriptionally silent" RXRα tetramer [37]. Linoleic and linolenic acids have also been described as potential endogenous ligands of RXR [38]. While the consensus on what constitutes the physiologically relevant endogenous RXR ligand(s) is currently lacking, it is clear that synthetic RXR agonists have a pharmacological value [39].

**Fig 1:**
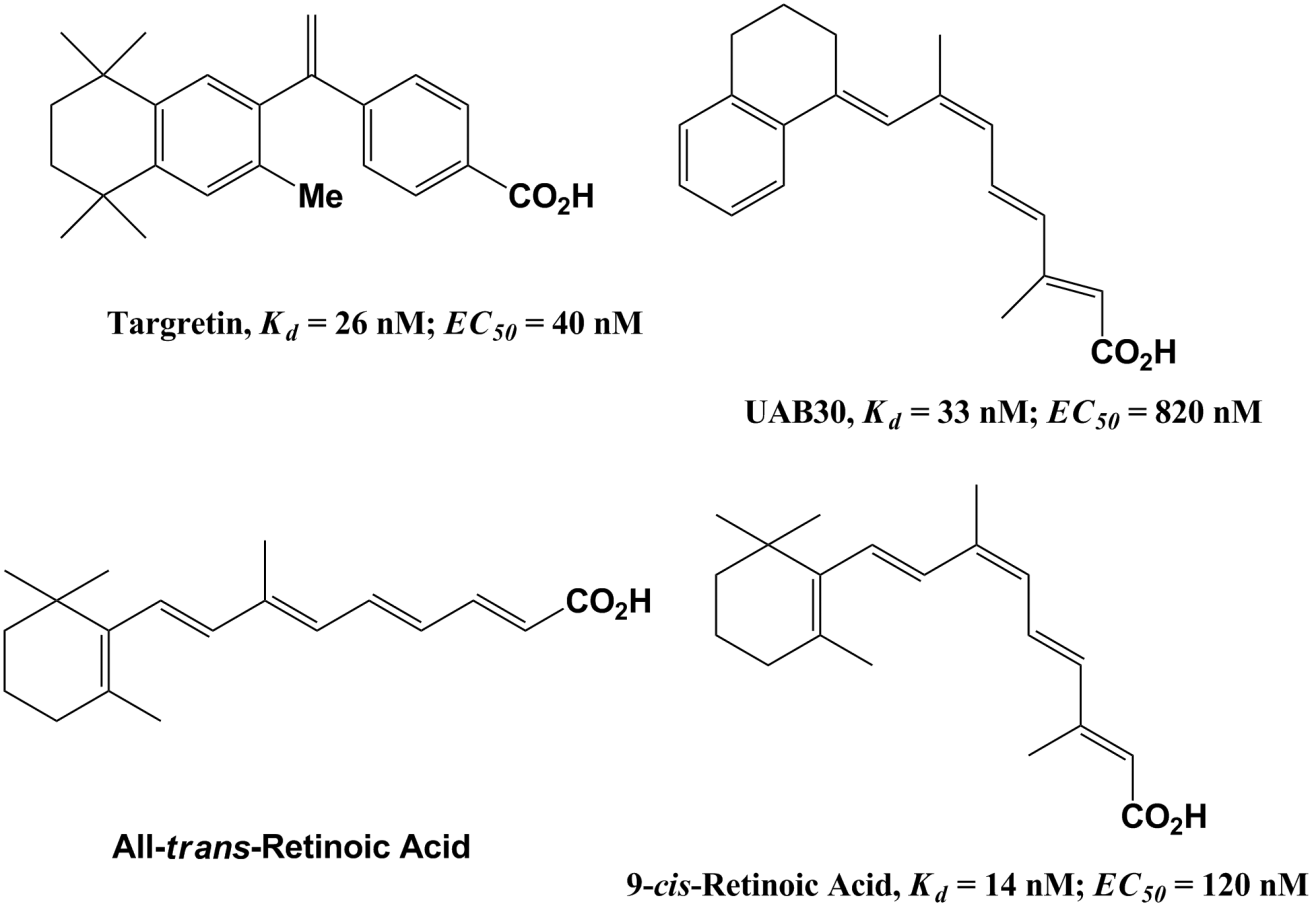
Chemical structures of RXR and RAR ligands. *K*_d_ values for 9-*cis*-RA and UAB30 are from Grubbs et al. [43] and for Targretin (Bexarotene) from Desphande et al. [44]. EC_50_ values are from Atigadda et al. [45].

The RXR-selective agonist, bexarotene (Targretin) (Fig 1), is approved clinically for the treatment of cutaneous T-cell lymphoma [40] and has demonstrated clinical efficacy in treatment/prevention of non–small-cell lung cancer [41, 42].

Human phase 1 trials revealed that bexarotene is better tolerated than ATRA and 9-*cis*-RA. However, oral use of bexarotene elevates serum triglycerides to a similar extent as observed for other clinically used retinoids (e.g., ATRA). This side effect is a major concern in the chronic, oral administration of these agents to a high-risk but otherwise healthy population [43, 46].

Previously we designed selective RXR agonists that were shown to be effective as inhibitors of mammary cancer formation but with lower toxicity than bexarotene [43, 47, 48]. One of these analogs (UAB30) did not increase serum triglycerides in rodents [43] and humans [46], but was nevertheless effective in preventing mammary cancers in rats. Like 9-*cis*-RA and bexarotene, UAB30 binds to human RXRα ligand binding domain in an L-shaped geometry, and binding of these agonists causes a nearly identical set of conformational changes on the ligand binding domain of human RXRα to recruit a coactivator peptide to the surface of the domain [49, 50]. While UAB30 clearly shows promise as a low-toxicity chemopreventive drug, the mechanism of UAB30 action at the molecular level is poorly understood. In this study, we investigated the biological effects of UAB30 on growth and differentiation of human keratinocytes using a model of human organotypic epidermis, and also examined the effects of UAB30 on gene expression in mouse skin from animals pre-treated with UAB30 before UVB irradiation. The results of this study provide evidence that the RXR specific rexinoid UAB30 acts by increasing levels of ATRA and, as a consequence, upregulates ATRA-responsive genes necessary for epidermal homeostasis.

## Materials and Methods

### 1. Preparation of organotypic skin rafts and treatment with UAB30 or bexarotene

Neonatal foreskins were obtained from the Newborn Nursery of the University of Alabama at Birmingham Hospital in compliance with University of Alabama at Birmingham Institutional Review Board (IRB) regulations. As determined by the institutional IRB, the use of discarded unidentifiable foreskin tissue met the requirements for an exemption from IRB approval. Epidermal raft cultures were prepared as described previously [51]. Briefly, primary human keratinocytes (PHKs) were isolated from freshly collected neonatal foreskins and cultured in DermaLife calcium-free medium (Lifeline Cell Technology, Walkersville, MD). PHKs were seeded onto a dermal equivalent consisting of collagen with embedded Swiss 3T3 J2 fibroblasts. After 3 days, skin equivalents were lifted onto stainless steel grids and cultured at the medium-air interface using raft culture medium prepared from Dulbecco's modified Eagle's medium, Ham's F12 medium, and bovine fetal serum, which was supplemented with cholera toxin, insulin, apo-transferrin, hydrocortisone-21, and human epidermal growth factor as described previously [51]. The raft culture medium was supplemented with either 2 μM UAB30 or 2 μM bexarotene (added as an ethanolic solution), as indicated, and applied from the day the skin equivalents were lifted onto the grids until harvest. The raft cultures were allowed to stratify and differentiate for 11 days, whereupon they were harvested for analysis.

To analyze changes in gene expression in response to retinol or ATRA, the fully grown skin rafts were incubated overnight with either 2 μM all-*trans*-retinol, 0.1 μM RA, or vehicle supplied in culture medium. Each treatment group included four rafts. Gene expression in skin rafts was analyzed by qPCR [51].

### 2. Detection of ATRA in skin rafts

Aliquots from a concentrated solution of UAB30 in DMSO (50 mM) were added to the culture medium to achieve a final concentration of 2 μM. The culture medium was replaced every other day, and supplemented with fresh UAB30. Medium for the control samples was supplemented only with DMSO. After harvesting, epidermis of raft cultures was peeled of underlying collagen beds. UAB30-treated or DMSO-treated cultures were pooled into three samples containing five rafts each, and retinoids were extracted essentially as described in [52]. Each sample was homogenized in 0.5 mL of ice-cold phosphate-buffered saline (PBS) in the dark, transferred to siliconized glass tube, and mixed with 0.5 mL of ethanol containing 0.025 M potassium hydroxide. Non-polar retinoids were extracted with 2 mL of hexane, organic phase was dried under the stream of nitrogen, reconstituted in 50 μL of hexane:acetonitrile (70:30), and analyzed by reverse phase HPLC as described before [52]. The remaining aqueous phase was acidified by the addition of 45 μL of 4 M hydrochloric acid, and polar retinoids (including ATRA and UAB30) were extracted with another 2 mL of hexane. The extract was dried and reconstituted in 400 μL of acetonitrile.

LC-MS-MS analysis of ATRA in tissue samples was carried out essentially as described before [53] with several modifications. To quantify the concentration of ATRA or UAB30 present in the dried extracts, 50-μL aliquots were injected into a Shimadzu LC-10AD HPLC (including a degasser) coupled to an Applied Biosystems 4000 Q Trap mass spectrometer. The column used for all analyses was a SUPELCOSIL ABZ PLUS (10 cm x 2.1 mm, 3 μm). Mobile phase A containing 40% acetonitrile, 30% methanol, and 30% ultra-pure water was mixed with mobile phase B containing 55% acetonitrile, 30% methanol, and 15% ultra-pure water using a gradient program. Each mobile phase contained 0.01% v/v of formic acid. The gradient program used for mixing the mobile phases was: 0 to 5 min, 100% A to 100% B; 5 to 19 min, 100% B; 19 to 20 min, 100% B to 100% A; 20 to 30 min, 100% A. The flow rate of the mobile phase was kept constant at 200 μL/min. The mass spectrometer was operated with an atmospheric pressure chemical ionization (APCI) source in multiple reaction monitoring controlled by the Analyst 1.4.2 software. The dwell time for ATRA and UAB30 was 40 ms. The conditions for optimum positive APCI detection were: curtain gas 10, nebulizer gas 3, collision gas 6, ion source 70, and temperature 350°C.

Each sample was injected three times, and the average of three injections was used to estimate its ATRA or UAB30 concentration. For ATRA, the 301 *m/z* was selected for in quadrupole 1 (Q1), and the 123 *m/z* ion fragment ions were quantified in quadrupole 3 (Q3). For UAB30, the 295 *m/z* was selected for Q1, and the 165 *m/z* was selected for Q3. Prior to analysis each peak was optimized for their declustering potential, entrance potential, collision energy, and collision cell exit potential using the optimization subroutine in Analyst.

To quantitate ATRA levels, a calibration curve was run 0.0–1.6 pmol/50 μL ATRA injection (7 concentrations varying 2-fold) using 3 injections for each concentration. The total ion current area (TIC) of the 123 *m/z* peak was fit to a linear equation to establish the calibration curve. The TIC area of the 123 m/z peak was measured three times and averaged. The endogenous concentration of ATRA in the samples was determined using the averaged peak area and the linear calibration curve. The same method was used for UAB30 except the 165 *m/z* fragment peak was used for the calibration curve and quantitation and a different range of concentrations was used in construction of the calibration curve (0.0–1.0 pmol/50 μL UAB30 injection).

### 3. H&E staining

The rafts were fixed in 10% buffered formalin, and embedded in paraffin. Paraffin-embedded skin rafts were cut into 5-μm sections, mounted on Superfrost/Plus slides (Fisher Scientific, Pittsburgh, PA), and then deparaffinized and processed for hematoxylin (Poly Scientific, Bay Shore, NY) and eosin (Fisher Scientific) staining as described previously [35]. All sections were analyzed at a 20×magnification using AxioImager A2 microscope equipped with an AxioCam camera and AxioVision image capture software (Carl Zeiss MicroImaging, Inc., Thornwood, NY).

### 4. QPCR analysis

For RNA extraction, epithelium was separated manually from the collagen bed and RNA was double-extracted using TRIZOL reagent (Invitrogen, Carlsbad, CA) according to the manufacturer’s instructions. Treatment by RQ1 RNase-free DNase (Promega, Madison, WI) at 37°C for 30 min was performed between the two extractions. The concentration of extracted RNA was determined using the Nanodrop ND-1000 spectrophotometer (Thermo Scientific). First-strand cDNA was synthesized from 3.0 μg of total RNA with Superscript III first-strand synthesis kit (Invitrogen, Carlsbad, CA) according to the manufacturer's protocol. First-strand cDNA was synthesized from 3.0 μg of total RNA with Superscript III first-strand synthesis kit (Invitrogen, Carlsbad, CA). Sequences of the primers are available upon request. Real-time PCR analysis was conducted on Roche LightCycler^®^480 detection system (Roche Diagnostics) with SYBR Green as probe (LightCycler^®^480 CYBR Green I Master, Roche, Indianapolis, IN). Relative gene expression levels were calculated using the comparative CT method by normalization to reference genes [54]. Three to four individual rafts were included in each qPCR experiment. To evaluate the significance of differences in expression levels of each transcript between control and drug-treated rafts, an unpaired *t* test was performed, and the two-tailed *p* value was determined using GraphPad InStat (version 3.00; GraphPad Software, San Diego, California).

### 5. Animal studies

Ptch1^+/-^/SKH-1 hairless mice (6–8 weeks old) used in this study were generated by crossing Ptch1^+/-^/C57BL/6 mice (Jackson Laboratory, Bar Harbor, ME) with SKH-1 hairless mice (Charles River Laboratories, Wilmington, MA) as described earlier [55]. The animals were housed under standard conditions of a 12 h dark/12 h light cycle, temperature of 24±2°C and relative humidity of 50±10%. Ptch1^+/-^/SKH-1 hairless mice were treated with placebo or UAB30 (40 mg/kg body weight; given orally twice a week one hour prior to UVB irradiation). Mice were chronically irradiated with UVB (180 mJ/cm^2^ twice weekly for 30 weeks) as described earlier [55, 56]. The animal studies and specific procedures (APN 110208363) described here were approved by the University of Alabama Institutional Animal Care and Use Committee (IACUC). Mice were euthanized as per IACUC recommendations. Their dorsal skin/tumors were harvested and banked for subsequent studies. Control skin was excised from the age-matched mice receiving no treatment. For this study, we used banked skin/tumor tissues from placebo or UAB30-treated mice.

## Results

**Fig 2:**
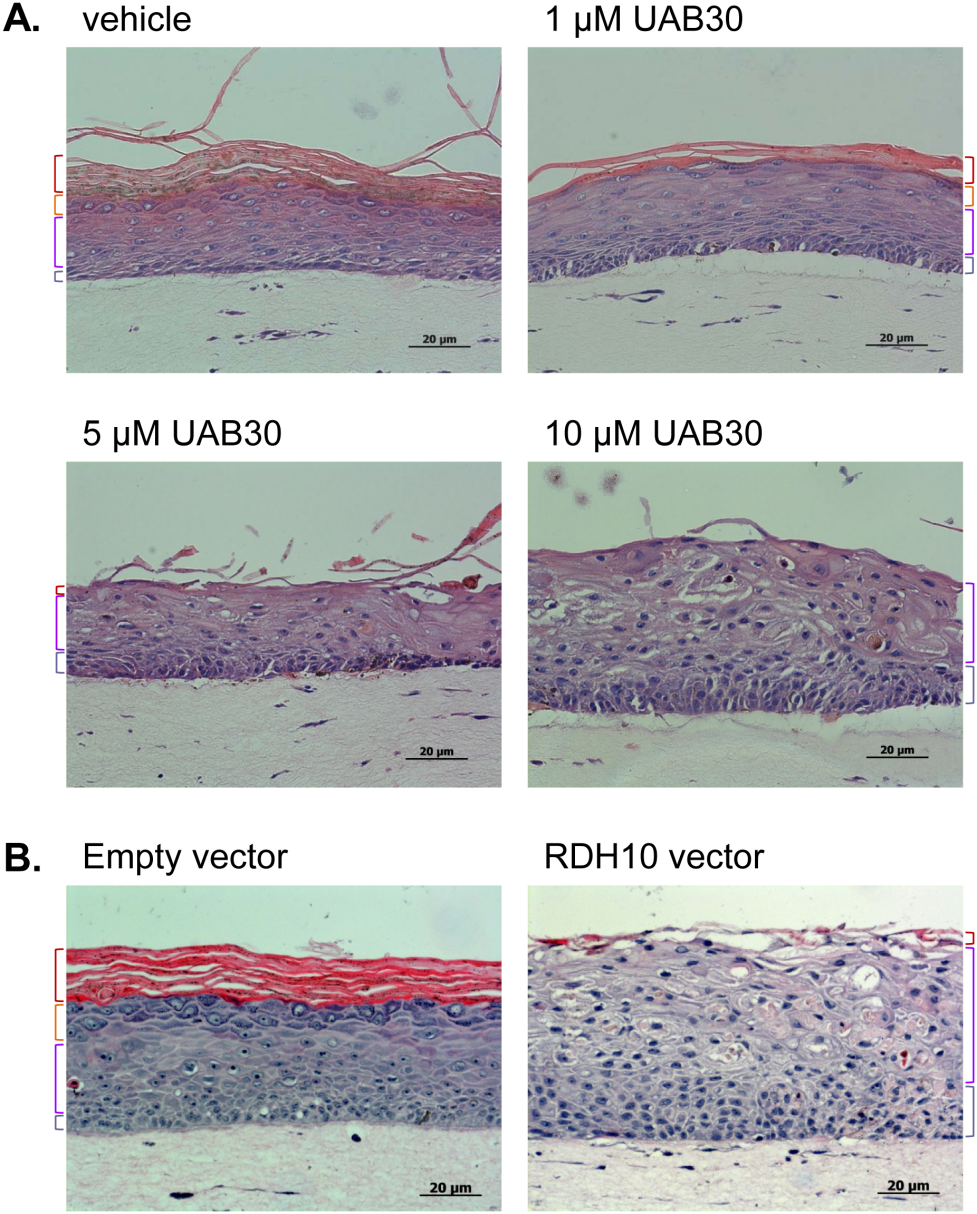
H&E staining of skin rafts treated with UAB30. **A**, Human organotypic skin raft cultures were grown in the presence of 0–10 μM UAB30. **B**, Primary neonatal keratinocytes (PHKs) were infected with Moloney murine leukemia retroviral vector pBabe puro expressing RDH10 and selected with 1.5 μg/ml puromycin for 2 days as described previously [51]. Retrovirus-transduced PHKs were cultured at the medium-air interface to produce RDH10 transgenic rafts. Differently colored brackets demarcate the layers of epidermis: cornified (red), granular (orange), spinous (purple), and basal (gray). Note the similarly increased thickness of the basal layer (gray bracket) and reduced differentiation to cornified layer (red bracket) in rafts treated with 10 μM UAB30 as compared with RDH10-overexpresing rafts.

To evaluate the biological effects of UAB30 on growing and differentiating human keratinocytes, UAB30 was added to the culture medium at the onset of organotypic skin raft formation. The rafts were grown in the presence of 0 to 10 μM UAB30 for 11 days. As PHKs proliferated and formed stratified epithelium, significant differences were noted in the appearance of rafts treated with various doses of UAB30 compared to rafts treated with vehicle. To evaluate the morphology of epidermis, rafts were sectioned and stained with H&E. This analysis revealed that control rafts had a well-developed histological appearance with clearly visible spinous, granular, and cornified layers (Fig 2A). In contrast, the granular layer in UAB30-treated rafts was developed poorly, and the cornified layer was progressively lost with increasing concentrations of UAB30 from 1 to 10 μM (Fig 2A). This pattern was strikingly similar to that observed in skin rafts overexpressing Retinol Dehydrogenase 10 (RDH10) (Fig 2B) [51], suggesting that treatment with UAB30 resulted in increased ATRA signaling through RXR/RAR heterodimers.

**Fig 3:**
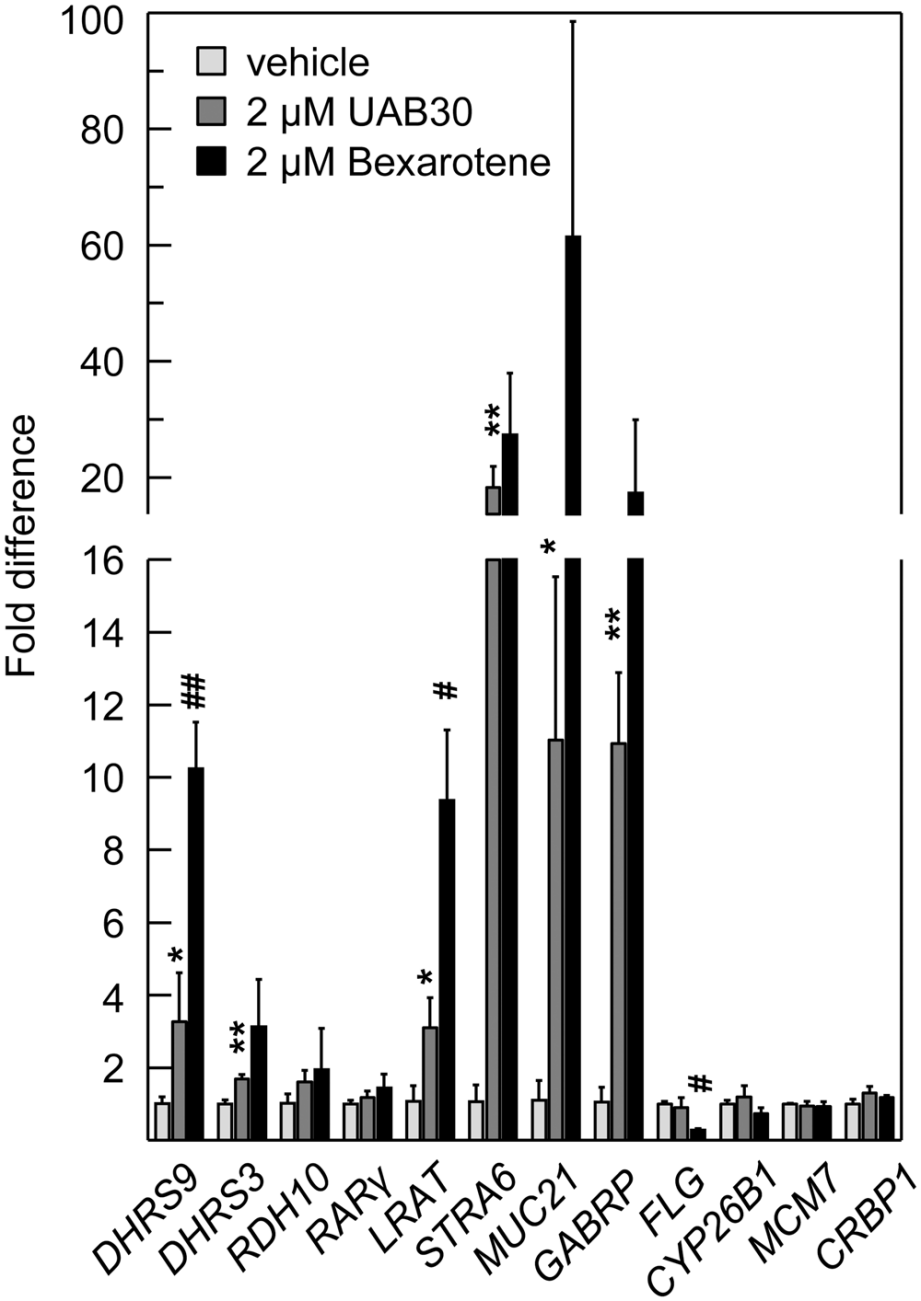
QPCR analysis of gene expression in skin rafts treated with UAB30 or Bexarotene. Three individual rafts were used for each treatment. QPCR analysis for each raft was done in triplicate. Data were normalized per peptidylprolyl isomerase A (*PPIA*, cyclophilin A) and represent average ± SEM. *DHRS9*, dehydrogenase/reductase member 9 (SDR9C4); *DHRS3*, dehydrogenase/reductase member 3 (SDR16C1, also known as retSDR1); *RDH10*, retinol dehydrogenase 10 (SDR16C4); *RARγ*, retinoic acid receptor γ; *LRAT*, lecithin retinol acyltransferase; *STRA6*, stimulated by retinoic acid gene 6; *MUC21*, mucin 21; *GABRP*, γ-aminobutyric acid (GABA) A receptor π; *FLG*, filaggrin; *CYP26B1*, cytochrome P450 26B1; *MCM7*, minichromosome maintenance complex component 7; *CRBP1*, cellular retinol binding protein type 1. Symbol * indicates statistically significant differences in expression of genes in UAB30-treated rafts *versus* vehicle-treated rafts. Symbol # indicates the differences in expression of genes in bexarotene-treated rafts *versus* UAB30-treated rafts. *and ^#^ indicate p values <0.05; ** and ^##^ indicate p values <0.005.

To obtain further evidence, we analyzed the expression levels of ATRA target genes in UAB30-treated rafts using qPCR analysis. For reference, this experiment included treatments of skin rafts with another RXR agonist, bexarotene, at the same concentration (2 μM) as UAB30. Importantly, to exclude the inter-individual donor response variability, treatments with UAB30 and bexarotene were conducted using the same batch of keratinocytes. QPCR analysis revealed that: first, UAB30 indeed up-regulated the expression of several well-established ATRA target genes (*DHRS3*, *LRAT*, *STRA6*); and second, that bexarotene upregulated some of the same genes with a greater potency than UAB30 (Fig 3).

The sensitivity and response to ATRA generally varies from gene to gene. Our previous study identified mucin 21 (*MUC21*) and γ-aminobutyric acid A receptor subunit π (*GABRP*) as the strongest responders to ATRA in human skin raft culture [51]. Consistent with these previous observations and as an additional confirmation of enhanced ATRA signaling, *MUC21* and *GABRP* were the most up-regulated transcripts in UAB30- and bexarotene-treated skin rafts at 2 μM concentrations of each compound, followed by *STRA6* (Fig 3).

**Fig 4:**
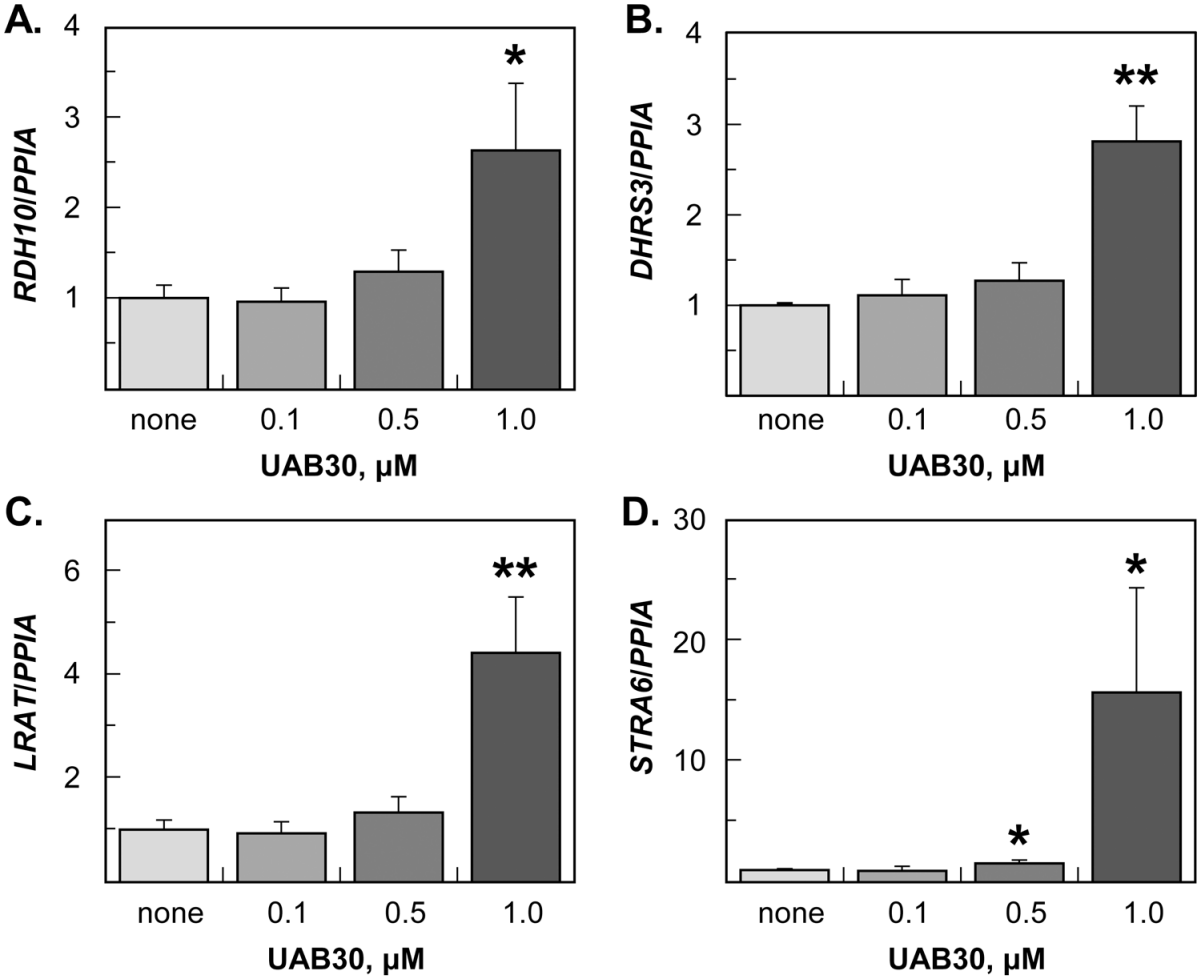
QPCR analysis of gene expression in skin rafts treated with different doses of UAB30. QPCR analysis was performed as described in Fig 3. Error bars represent mean ± SEM of three independent rafts. *p<0.05; **p<0.01.

To determine whether the effect of UAB30 on gene transcription was dose-dependent, we treated skin rafts with three different concentrations of UAB30. Most of the ATRA-sensitive genes tested in this study required at least 1 μM UAB30 to respond, except for *RARγ*, which showed upregulation already at 0.1 μM UAB30 (S1 Fig). The expression of filaggrin in skin rafts treated with 1 μM UAB30 was decreased by ~2-fold (S1 Fig), in agreement with the reduced number of cornified layers (Fig 2). The expression of PPARα target genes (*SREBP1c* and *SREBP2*) mediated by RXR/PPAR heterodimers was only weakly affected by 1 μM UAB30 treatment (S1 Fig), suggesting that, at least in human epidermis, the PPARα pathway was not a major target of UAB30 action. Importantly, this experiment demonstrated that UAB30 dose-dependently upregulated the expression of genes encoding enzymes responsible for the maintenance of tissue levels of retinol and retinyl esters (LRAT, STRA6) and ATRA biosynthesis (RDH10, DHRS3) (Fig 4).

**Fig 5:**
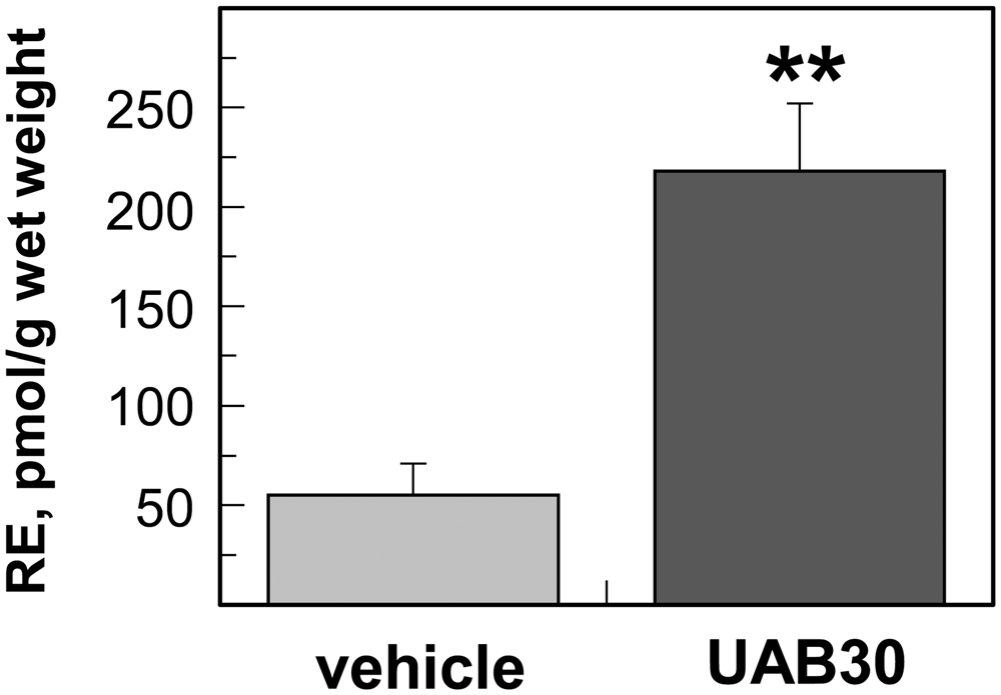
HPLC analysis of retinyl esters in UAB30-treated skin rafts. Retinyl esters were extracted from three samples each containing five pooled UAB30-treated or DMSO-treated skin rafts. Data represent mean ± SEM; **p<0.01.

To determine whether the UAB30-mediated increase in expression of retinoid metabolic genes resulted in altered retinoid homeostasis, skin rafts were grown in media supplemented with vehicle (DMSO) or 2 μM UAB30. Following extraction from skin rafts, retinol and retinyl esters were separated and quantified by HPLC, whereas ATRA levels were measured by the more sensitive LC-MS-MS detection method. HPLC analysis showed that treatment with UAB30 resulted in a ~4-fold increase in retinyl esters in UAB30-treated rafts (218 ± 34 pmol/g wet weight), relative to rafts treated with DMSO (55 ± 16 pmol/g wet weight) (Fig 5). The significant increase in retinyl esters was in agreement with the observed upregulation of *STRA6* and *LRAT* expression.

To determine the background levels of ATRA in control DMSO-treated skin rafts, ATRA was measured using the LC-MS-MS method in three pooled samples each containing five rafts. Each sample was injected three times. The mass spectrum of the fragments from Q3 was examined for the presence of the 123 *m/z* fragment peak, which is the most intense daughter peak from ATRA fragmentation of the 301 *m/z* parent ion peak. The limit of quantitation (LOQ) was defined by a peak with a height 2 times the signal-to-noise. From nine injections of these samples, the 123 *m/z* peak was not detectable in eight of the total ion count (TIC) chromatograms; for one injection, the 123 *m/z* peak was detectable at a level below the LOQ. Based on these measurements, we concluded that ATRA concentration in vehicle-treated skin raft samples was below the LOQ (samples DMSO-1-DMSO-3) (Table 1).

**Table 1:** ATRA concentration in samples of skin rafts.

Sample	Wet weight of the extracted tissue, mg	ATRA concentration in tissue, pmol/g	ATRA concentration in tissue, ng/g
**DMSO-1**	88	Below LOQ	Below LOQ
**DMSO-1**	57	N.D.S.	N.D.S.
**DMSO-1**	75	N.D.S.	N.D.S.
**UAB30-1**	39	23	6.9
**UAB30-1**	71	19	5.7
**UAB30-1**	50	20	6.0

To determine the levels of ATRA in skin rafts treated with 2 μM UAB30, three pooled samples each containing five UAB30-treated rafts, were analyzed using the same LC-MS-MS method. As before, each sample was injected three times. The mass spectrum of the fragments from Q3 was examined for the presence of the 123 *m/z* fragment peak. For every injection, the 123 *m/z* peak was detectable above the LOQ in the TIC chromatograms. The TIC areas were averaged and compared to the ATRA calibration curve. All UAB30-treated raft samples contained detectable ATRA. Sample UAB30-1 had lesser amount of total tissue than samples UAB30-2 and UAB30-3 (Table 1). The concentration of ATRA in UAB30-1 was at the LOQ and was not used in further calculations of ATRA levels. In samples UAB30-2 and UAB30-3, ATRA concentration reliably exceeded the LOQ (Table 1). An average of these two samples gave an estimated 19.5 pmol/g (19.5 nM) of ATRA in UAB30-treated rafts. Thus, LC-MS-MS analyses provided direct evidence that treatment with UAB30 resulted in a substantial increase in epidermal ATRA levels relative to rafts treated with DMSO vehicle.

Each sample contained five pooled skin rafts, treated either with vehicle (DMSO) or with 2 μM UAB30. Extracted samples were reconstituted in 400 μL of acetonitrile. Fifty microliters were injected onto the LC-MS-MS three times. The peak areas of the 123 m/z fragment from three injections were averaged and compared to a standard curve of ATRA. The limit of quantitation (LOQ) was defined by a peak with a height 2 times the signal-to-noise. When no peak was observed above the baseline peak-to-peak noise, no detectable signal (N.D.S) is indicated. When a peak was observed above peak-to-peak noise but below the LOQ, Below LOQ is indicated.

To determine UAB30 levels in skin rafts, the 165 m/z peak was measured for each run. As expected the 165 m/z peak was absent for each DMSO-treated raft and clearly present for each UAB30-treated raft. Using external calibration method, we determined that, at 2 μM UAB30 in culture medium, the actual concentration of UAB30 achieved in skin raft tissue was 1.3±0.3 μM. This concentration is above the reported EC_50_ value (0.82 μM, Fig 1) for UAB30-mediated activation of RXRα as determined using a luciferase reporter system in the HEK293 cell line transiently transfected with a Gal4 reporter construct containing the hRXRα-LBD [45].

**Fig 6:**
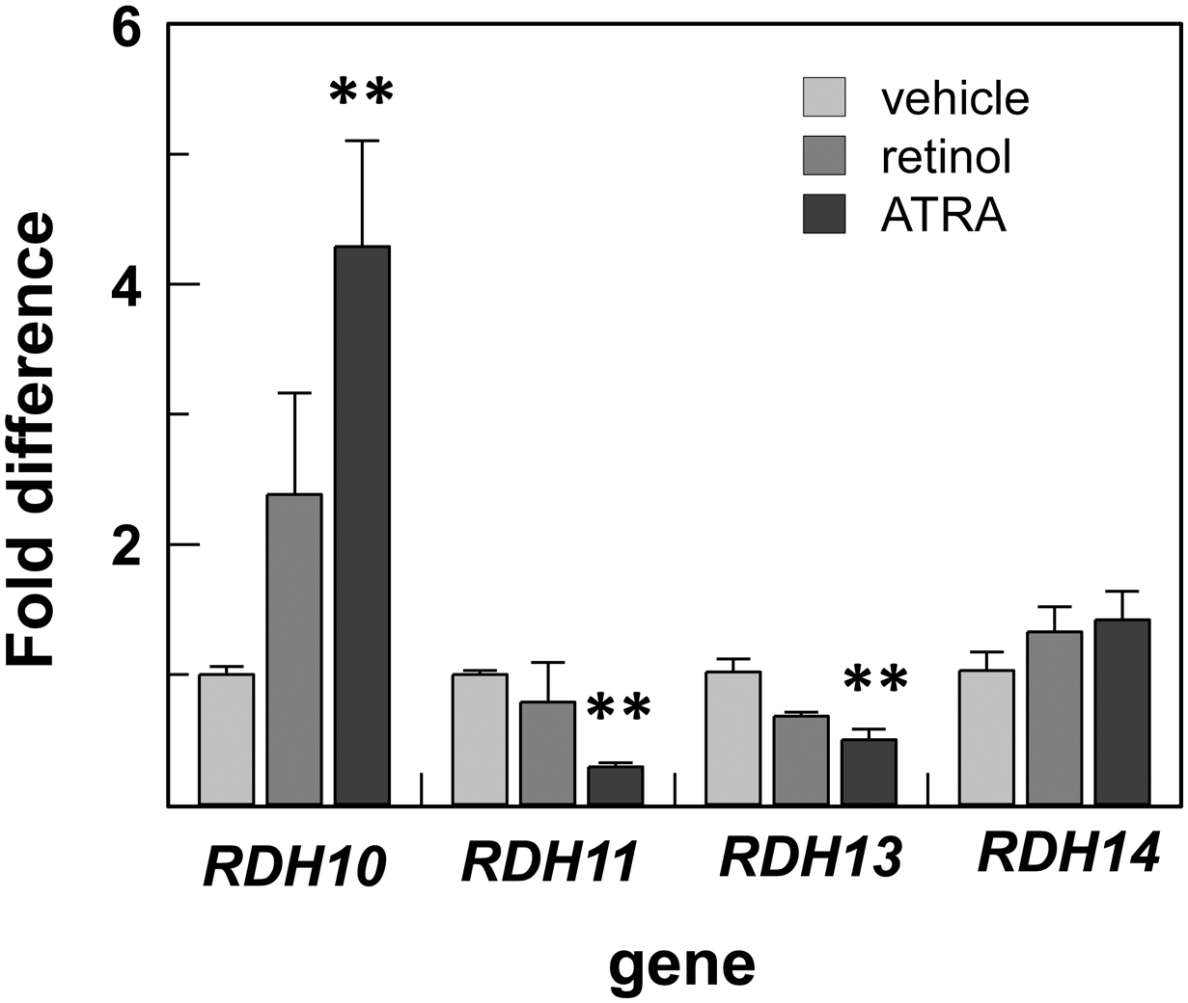
UAB30 dose-dependent induction of retinoid metabolic genes. QPCR analysis was performed as described in Fig 3. Error bars represent mean ± SEM of three independent rafts. *p<0.05; **p<0.01.

*LRAT*, *STRA6*, and *DHRS3* genes are well-established targets of ATRA signaling. However, our finding that *RDH10* expression was also upregulated in UAB30-treated rafts was unexpected. To determine whether *RDH10* is responsive to ATRA in human skin epidermis, we supplemented skin raft culture medium with 2 μM retinol or 0.1 μM ATRA and examined the levels of *RDH10* transcripts by qPCR. This analysis showed that both retinol and ATRA induced the expression of *RDH10* in human epidermis (Fig 6). Interestingly, *RDH10* was also induced by ATRA in HEK293 cells, but not in HepG2 cells (data not shown), suggesting that the ATRA-mediated induction of *RDH10* is cell-type specific. In contrast to *RDH10*, the genes encoding enzymes that function as retinaldehyde reductases when expressed in whole cells (*RDH11*, *RDH13*, and *RDH14*) [6] were either downregulated or unchanged by the treatment of human epidermis with ATRA or retinol. A decrease in the total cellular retinaldehyde reductase activity could further contribute to the elevated levels of ATRA and enhanced ATRA signaling.

**Fig 7:**
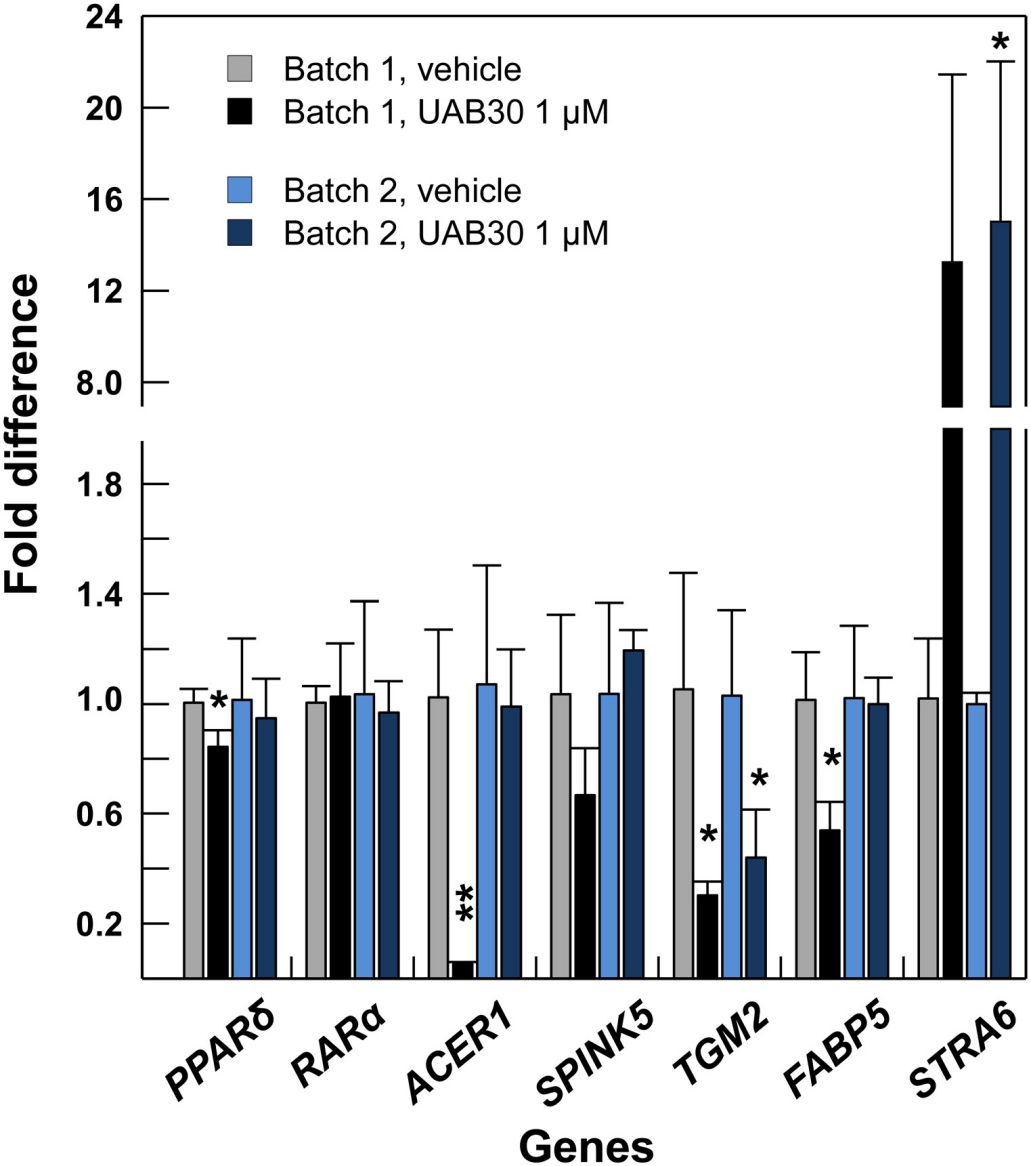
QPCR analysis of select RXR target genes in skin rafts treated with UAB30. QPCR analysis was performed as described in Fig 3 using skin rafts prepared from two different batches (designated 1 and 2) of skin keratinocytes each pooled from 2–3 donors. Error bars represent mean ± SEM of three independent rafts. *p<0.05; **p<0.005.

It has been reported that ATRA can bind not only to RARs but also to PPARδ, albeit with a higher *K*_d_ value than to RAR [57], and that the ratio between cellular retinoic acid binding protein type II (CRABP-II) and fatty acid binding protein type 5 (FABP5), the two carrier proteins which deliver ATRA to RAR and PPARδ, respectively, controls the partitioning of ATRA [58]. Signaling through PPARδ is important for maintenance of skin permeability-barrier integrity [59, 60]. To explore whether the increased concentration of ATRA in UAB30-treated skin rafts resulted in activation of PPARδ signaling pathway, we examined the expression levels of *FABP5*, *PPARδ*, *RARα*, and several markers of permeability barrier such as alkaline ceramidase 1 (*ACER1*), serine protease inhibitor Kazal type-5 **(***SPINK5*), and transglutaminase 2 (*TGM2*). We used skin rafts prepared from two different batches of human keratinocytes, which were pooled from 2–3 individual donors. As shown in Fig 7, treatment with UAB30 resulted in little or no change in the expression of *RARα*, whereas the transcript levels of *PPARδ*, *FABP5*, *ACER1*, and *SPINK5* were somewhat reduced in batch 1 of skin rafts but not in batch 2. The expression of *TGM2* was significantly reduced in skin rafts from both batches. At the same time, *STRA6* transcript was consistently and strongly upregulated. These data argued against the activation of *PPARδ* signaling in UAB30-treated skin rafts. The observed downregulation of *ACER1* and *TGM2* was consistent with the decreased numbers of suprabasal differentiated layers and the reduction in cornified layers in UAB30-treated skin rafts.

**Fig 8:**
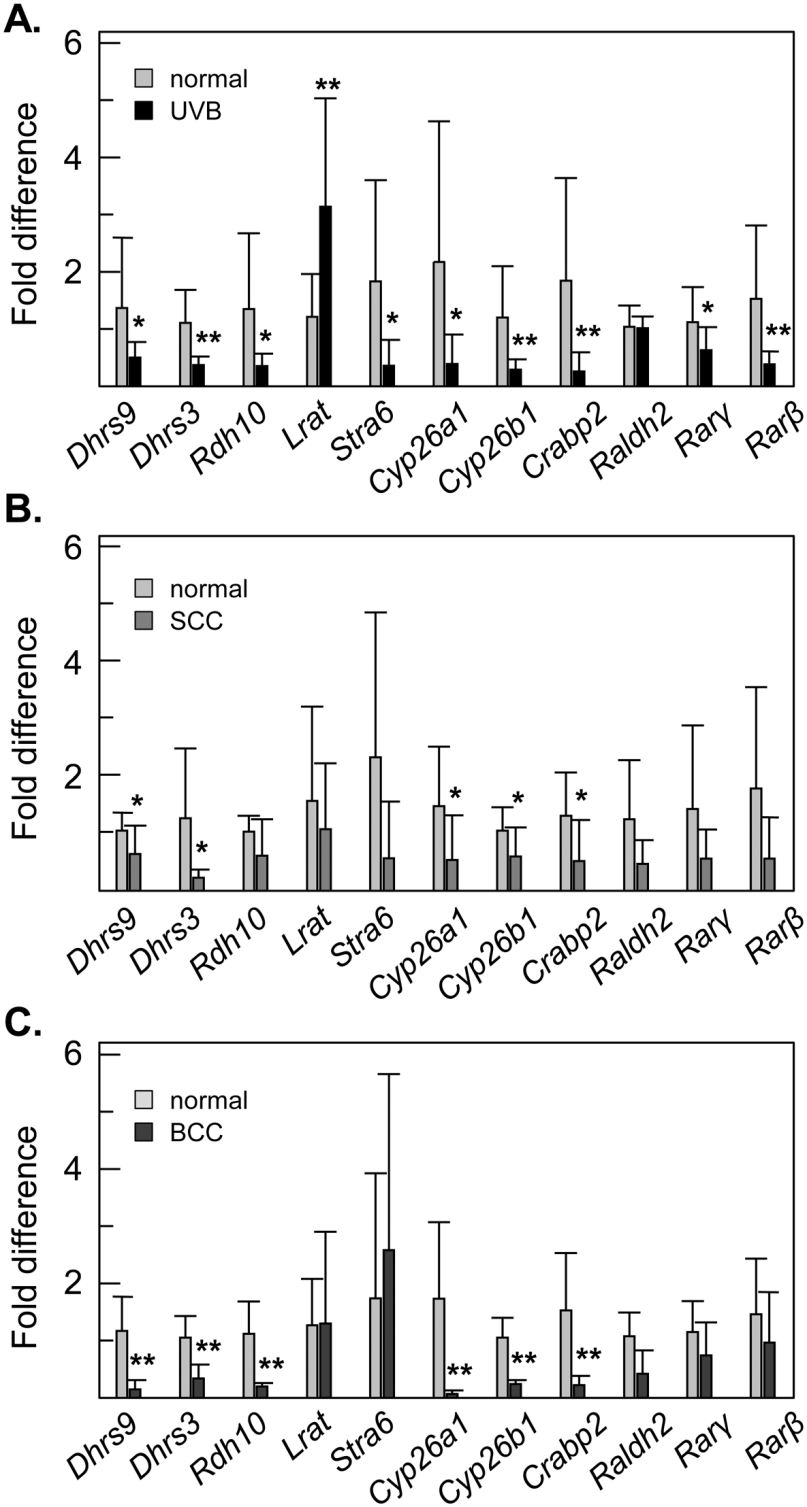
Expression levels of ATRA target genes in UVB irradiated mouse skin and in mouse models of UVB-induced SCC and BCC. **A.** UVB irradiation. **B.** SCC. **C.** BCC. QPCR analysis was performed as described in Fig 3. Gene expression was normalized per the most stable gene in each model (*Hprt* for UVB; *Gapdh* for SCC; actin for BCC). Normal mice, n = 13; UVB-irradiated, n = 13; SCC and BCC, n = 7. *p<0.05; **p<0.005.

Solar ultraviolet B (UVB) radiation has been shown to induce inflammation, DNA damage, p53 mutations and alterations in signaling pathways eventually leading to skin cancer. To determine whether ATRA signaling is affected by UVB irradiation and carcinogenesis in mouse models, we performed qPCR analysis of skin samples from UVB-irradiated mice and from mouse models of UVB-induced squamous cell carcinoma (SCC) and basal cell carcinoma (BCC). UVB irradiation caused a statistically significant decrease in expression of nine genes out of eleven that were analyzed (Fig 8A). These included well-known ATRA target genes: *Dhrs3*, *Cyp26a1*, *Cyp26b1*, *Rarγ*, *Rarβ*, *Crabp2*, and *Stra6*. Several of the same ATRA-sensitive genes were downregulated in mouse models of UVB-induced SCC and BCC (Fig 8B and 8C). Surprisingly, the mouse *Lrat* gene appeared to be upregulated by UVB irradiation, but did not show significant changes in expression in SCC or BCC. Importantly, the transcript encoding the mouse ortholog of RDH10, the enzyme that was shown to be primarily responsible for the biosynthesis of ATRA in human epidermis [51], was downregulated in mouse epidermis by UVB and in BCC, whereas in SCC it showed a tendency towards decreased expression.

**Fig 9:**
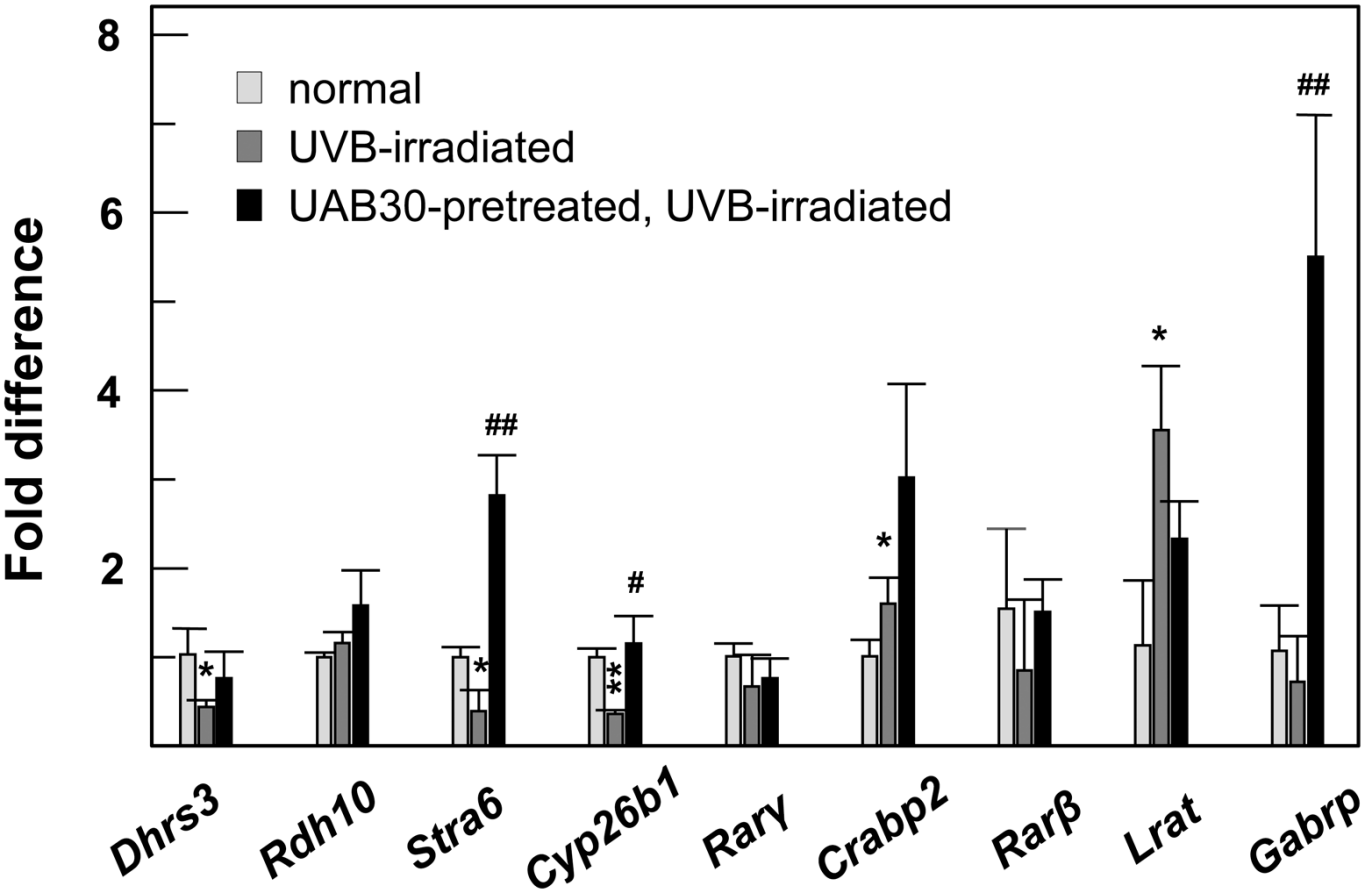
QPCR analysis of gene expression in UVB-exposed mice pretreated with UAB30. The mRNA from three individual animals was used for each condition. QPCR analysis for each mouse was performed in triplicates. Data were normalized per *Gapdh* and represent average ± SEM. Symbol * refers to expression of genes in epidermis of control *versus* UVB-exposed mice. Symbol # refers to expression of genes in epidermis of UVB-exposed mice *versus* UAB30-pretreated and UVB-exposed. *,^#^p <0.05; **,^##^p <0.01.

To determine whether treatment with UAB30 could protect ATRA signaling from the effects of UVB irradiation, we examined the levels of ATRA-sensitive transcripts in skin of mice receiving topical treatment with UAB30-containing cream prior to their exposure to UVB radiation (Fig 9). Control groups included mice receiving treatment with a vehicle and mice receiving UVB exposure without UAB30 pre-treatment. Consistent with the results of the previous experiment (Fig 8), UVB exposure led to a decreased expression of ATRA-sensitive *Dhrs3* (p = 0.03), *Stra6* (p = 0.02), and *Cyp26b1* (p = 0.0005) (Fig 9, gray bars). However, this decrease did not occur when the skin was pre-treated with UAB30 (Fig 9, black bars). In fact, the expression of *Stra6* gene was increased by UAB30 treatment by 2.8-fold relative to unexposed skin (p = 0.002). Remarkably, the mouse *Lrat* gene was again an exception, being upregulated by UVB irradiation (Fig 9). Treatment with UAB30 appeared to prevent the UVB-induced upregulation of *Lrat*. Due to limited number of samples (n = 3) several genes did not show a statistically significant decrease in expression upon UVB irradiation; however, some of these ATRA-sensitive genes were upregulated as a result of pre-treatment with UAB30 prior to UVB exposure when compared to unexposed controls (*Gabrp*, p = 0.01; *Crabp2*, p = 0.03). These results suggested that, in both human and mouse skin epidermis, UAB30 acted to enhance ATRA signaling and could protect ATRA-sensitive genes against UVB-induced downregulation.

## Discussion

RXR agonists can have pleiotropic effects on gene transcription because, in addition to RARs, RXRs form heterodimers with numerous other nuclear transcription factors [6]. In livers of rats, UAB30 was shown to activate genes associated with the aryl hydrocarbon (Ah) receptor, but minimal changes were observed in genes responsive to RXR heterodimers [61]. The results of this study suggest that, in human epidermis, UAB30 specifically targets the genes regulated by RXR/RAR heterodimers.

One possible mechanism for upregulation of ATRA-sensitive genes by UAB30 is by enhancing the potency of cellular ATRA, because together RAR and RXR agonists can exert a greater effect than ATRA alone [5]. However, our data indicate that, in addition to potential enhancement of transcriptional activity of the available ATRA, treatment with UAB30 leads to an overall increase in the total levels of ATRA in the cells. As shown by LC-MS-MS analysis, the concentration of ATRA in UAB-treated skin rafts is ~20 nM, whereas in DMSO-treated rafts the ATRA concentration is below the limit of quantitation (≤9 nM). These values agree well with previous estimates of the ATRA concentration in the epidermal cells [62].

The increase in ATRA is consistent with the observed upregulation of RDH10, a highly potent retinol dehydrogenase that contributes to ATRA biosynthesis in human epidermis [51]. Because RDH10 catalyzes the rate-limiting step, the oxidation of retinol to retinaldehyde, in the pathway of ATRA production, a ~2-fold increase in RDH10 expression levels induced by 1–2 μM UAB30 is likely to result in higher levels of ATRA. Furthermore, the upregulation of RDH10 is accompanied by the increased expression of retinol transporter STRA6 and LRAT, the enzyme responsible for generation of storage forms of retinol, retinyl esters. Indeed, HPLC analysis confirms that the upregulation of STRA6 and LRAT is translated into a 4-fold higher levels of retinyl esters in skin rafts treated with UAB30 compared to vehicle (DMSO) treated rafts. The larger pool of retinyl esters ensures that a stable supply of retinol is available to support the increased activity of RDH10 for conversion of retinol to ATRA.

Currently, very little is known about the factors that regulate the expression of RDH10, and there seems to be a difference in the regulation of this gene among different species. For example, in *Xenopus laevis*, rdh10 expression is suppressed by ATRA [63], but in chick, RDH10 is not affected by the excess or absence of ATRA [64]. As shown in the present study, treatment with either ATRA or retinol increases the level of RDH10 mRNA in human skin rafts. Thus, in human epidermis, RDH10 expression appears to be regulated by the RXR/RAR heterodimers. It is conceivable that UAB30 acts first by binding to RXR and potentiating the RXR/RAR-mediated transcriptional activity of available ATRA, followed by further rise in cellular ATRA as a result of the increased expression of RDH10, LRAT and STRA6.

Another group recently reported that topical application of selective RXR agonist LG268 from Ligand Pharmaceuticals increased ATRA levels in skin of mice and caused mild epidermal hyperproliferation [65]. ATRA concentration in murine skin after two weeks of topical treatment with LG268 was 25 ng/g (83 pmol/g) compared to skin treated with acetone (2.5 ng/g or 8.3 pmol/g). The authors speculated that the increase in ATRA was associated with the 2.4-fold upregulation of retinaldehyde dehydrogenase *Aldh1a2* (also known as *Raldh2*). Notably, the expression of *Rdh10* in these experiments seemed to be downregulated by LG268 (0.66-fold), and by ATRA (0.7-fold). Somewhat contradictory to the effect of ATRA, RARγ agonist was found to upregulate *Rdh10* expression (1.5-fold) in this study. In our skin raft model, 1 μM UAB30 did not appear to induce human *RALDH2* expression, but *RDH10* showed a reproducible increase in expression upon treatment with UAB30, and this increase occurred in a dose-dependent manner. Considering that the retinol dehydrogenase activity represents the rate-limiting step in ATRA biosynthesis, it seems befitting that the increase in ATRA in human rafts correlated with the increase in *RDH10* rather than *RALDH2* expression. However, the observed differences between mouse and human epidermis could be due to species-specific factors.

ATRA is absolutely essential for the maintenance of healthy skin epithelium, but the range of acceptable ATRA levels in the skin is remarkably narrow, and it is strictly controlled [62, 66]. Concentrations that exceed the optimal range suppress differentiation and promote hyperproliferation, while concentrations below this range lead to formation of orthokeratotic epithelium [66]. It has been demonstrated that the topical retinoid signal is transduced by RXRα/RARγ heterodimers in suprabasal keratinocytes, which, in turn, stimulate proliferation of basal keratinocytes *via* a paracrine signal [67]. The increased thickness and the loss of fully differentiated cornified layers observed in UAB30-treated rafts provide further evidence for the increased levels of ATRA and enhanced signaling through RXRα/RARγ heterodimers.

Numerous studies demonstrate that vitamin A deficiency leads to an increased development of spontaneous and chemically induced tumors [68]. On the other hand, dietary vitamin A supplementation appears to decrease chemically induced tumor incidence. ATRA prevents tumor development by inhibition of proliferation [69–71], stimulation of differentiation [72], induction of apoptosis [73, 74] or combinations of these mechanisms. Our study demonstrates that the expression of ATRA sensitive genes is altered in UVB irradiated mouse skin and in mouse models of UVB-induced basal cell carcinoma and squamous cell carcinoma. These observations suggest that exposure to UVB disrupts retinoid metabolism and impairs ATRA signaling. The majority of ATRA sensitive genes are downregulated by UVB irradiation. However, the expression of *Lrat* in mouse UVB-exposed skin is upregulated. Previous studies have shown that LRAT expression and activity is reduced in various cancer cell lines and cancer tissues, including prostate, colorectal, breast, oral cavity, bladder, kidney and skin [75–79]. At the same time, very high, ectopic LRAT expression in oral epithelial basal cells makes these cells more sensitive to carcinogen induced tumorigenesis [80]. Thus, either too much or too little of LRAT seems to be equally harmful. In this respect, the upregulation of LRAT in UVB-exposed skin is consistent with reduced ATRA biosynthesis, because as reported previously, at high expression levels, LRAT can compete with RDH10 for retinol as substrate [81]. Interestingly, the expression of *Lrat* in mouse UVB-irradiated skin is reduced by treatment with UAB30, whereas the expression of *Rdh10*, *Stra6* and several other ATRA sensitive genes tends to be upregulated. Collectively, these data suggest that treatment with UAB30 normalizes the signaling mediated by RXR/RAR heterodimers by correcting the levels of retinoid metabolic enzymes and proteins that are altered by UVB irradiation.

Organ-transplant recipients are at a higher risk of developing UVB-induced non-melanoma skin cancers, because of the drugs required to maintain their grafted tissue. These medications suppress the host immune response that has evolved to protect against the growth and development of non-melanoma skin cancers. They also promote epithelial-mesenchymal transition by activating the TGFβ signal transduction pathway in epidermal keratinocytes [82–84]. As shown in this study, bexarotene (Targretin), the only rexinoid approved by the FDA, induces the same panel of ATRA-sensitive genes as UAB30, but with a greater potency. Unfortunately, hyperlipidemia is one of the dose-limiting toxicities of bexarotene in humans. Genomic, proteomic and metabolomic studies indicate that bexarotene induces triglyceride synthesis by hyper-stimulating transcription of genes under the control of the RXR/LXR heterodimer in rat livers [61, 85–87].

UAB30 was shown to prevent mammary cancers *in vivo* and is currently being evaluated in human Phase I clinical trials by the NCI. UAB30 also prevents the formation of squamous cell carcinoma (SCC) in Kruppel-like factor 4 (KLF4) transgenic mouse model [88], and inhibits tumor growth and increases survival in a murine neuroblastoma xenograft model [89]. The results of this study provide the first evidence that pre-treatment of mouse skin with UAB30 prevents changes in ATRA signaling induced by UVB irradiation by upregulating the uptake and conversion of retinol to ATRA. Extensive studies demonstrate that the effects of UAB30 are tissue-specific, and UAB30 does not act as an RXR agonist in liver [61]. Even though the potency of UAB30 is about 4-fold less than that of bexarotene, the lack of lipid toxicity makes it an attractive rexinoid for chronic administration to a high-risk population. Further studies are necessary to fully understand the mechanism of UAB30 action in skin in order to develop more potent and safe rexinoids.

## Supporting Information

S1 Fig QPCR analysis of gene expression in skin rafts treated with different doses of UAB30. QPCR analysis was performed as described in Fig 3. Error bars represent mean ± SEM of three independent rafts. *RARγ*, retinoic acid receptor γ; *MUC*, mucin 21; *GABRP*, γ-aminobutyric acid (GABA) A receptor π; *FLG*, filaggrin; *Srebp1c*, *Srebp2*, sterol regulatory element-binding protein 1c and 2.*p<0.05; **p<0.01.
(PPTX)
