# Supplementary material for: Retinoid X Receptor Agonists Upregulate Genes Responsible for the Biosynthesis of All-Trans-Retinoic Acid in Human Epidermis
Source: PLoS One. 2016 Apr 14;11(4):e0153556. doi: 10.1371/journal.pone.0153556 (PMC4831765; doi:10.1371/journal.pone.0153556)
Supplement: S1 Fig — QPCR analysis was performed as described in Fig 3. Error bars represent mean ± SEM of three independent rafts. RARγ, retinoic acid receptor γ; MUC, mucin 21; GABRP, γ-aminobutyric acid (GABA) A receptor π; FLG, filaggrin; Srebp1c, Srebp2, sterol regulatory element-binding protein 1c and 2.*p<0.05; **p<0.01. (PPTX) [file pone.0153556.s001.pptx]

## Slide 1
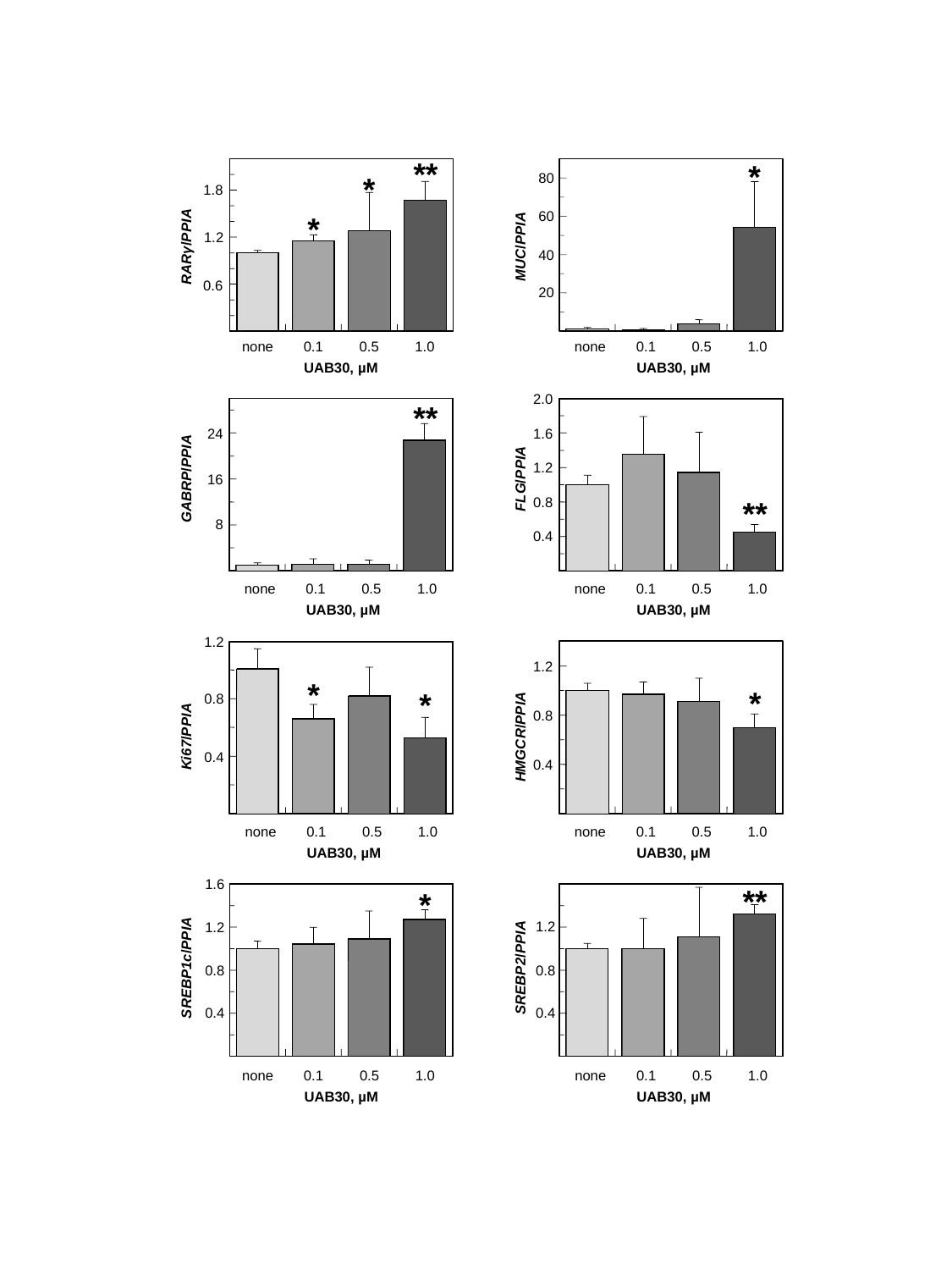

**
*
1.8
1.2
0.6
none
0.1
0.5
1.0
UAB30, µM
RARγ/PPIA
80
60
40
20
none
0.1
0.5
1.0
UAB30, µM
MUC/PPIA
2.0
1.6
1.2
0.8
0.4
none
0.1
0.5
1.0
UAB30, µM
FLG/PPIA
24
16
8
none
0.1
0.5
1.0
UAB30, µM
GABRP/PPIA
1.2
0.8
0.4
none
0.1
0.5
1.0
UAB30, µM
Ki67/PPIA
1.2
0.8
0.4
none
0.1
0.5
1.0
UAB30, µM
HMGCR/PPIA
1.6
1.2
0.8
0.4
none
0.1
0.5
1.0
UAB30, µM
SREBP1c/PPIA
1.2
0.8
0.4
none
0.1
0.5
1.0
UAB30, µM
SREBP2/PPIA
*
*
**
**
*
*
*
**
*
